# Immunologic Combinations in Multiple Myeloma: Synergistic Stimulation with T cells and NK cells

**DOI:** 10.7150/ijbs.127871

**Published:** 2026-04-16

**Authors:** ZhaoYun Liu, Jiao Lai, Chun Yang, Hui Liu, Kai Ding, Jia Song, Rong Fu

**Affiliations:** Department of Hematology, Tianjin Medical University General Hospital, 154 Anshan Street, Heping District, Tianjin 300052, China, Tianjin Key Laboratory of Bone Marrow Failure and Malignant Hemopoietic Clone Control, Tianjin Institute of Hematology, State Key Laboratory of Experimental Hematology.

**Keywords:** Multiple myeloma, Immunotherapy, Cancer Vaccines, bone marrow microenvironment

## Abstract

Bone marrow plasma cells proliferate malignantly in multiple myeloma (MM), a hematological cancer. MM is now the second most common hematologic illness, followed by non-Hodgkin's lymphoma. The tumor microenvironment (TME) is a crucial factor in MM development. T cells, natural killer (NK) cells, and natural killer T (NKT) cells are the key anti-MM immune cells in the bone marrow microenvironment (BMME). By direct contact or cytokine production, these cells prevent MM cells from proliferation and survival. However, a major factor contributing to the insufficiency of anti-tumor immune responses is the inhibition of T cells and NK cells functions. We aimed to summarize the mechanisms of T cells and NK cells suppression in MM and discuss emerging immunotherapies that target both cell types

## Introduction

MM is a malignancy originating from bone marrow plasma cells, and the onset and progression of MM are closely related to the BMME 1. The development of immunosuppressive cells, malfunctioning of effector cells, and inhibition of anti-tumor immunity through the production of cytokines and metabolites gradually disrupt the immune milieu, resulting in carcinogenesis, and progression 2, 3. The milieu surrounding myeloid-derived suppressor cells (MDSCs) in the bone marrow is constituted of complex interactions between extracellular matrix (ECM) proteins and a wide range of cellular constituents, such as endothelial cells, stromal cells, osteoblasts, and osteoclasts, as well as multiple immune cells, such as T cells, NK cells, monocytes, and MDSCs. These cells work together to promote the development of MDSCs 4. Immune cells, particularly T cells and NK cells, are essential to coordinate anti-tumor immune responses within the BMME 5. However, the tumor microenvironment in MM significantly inhibits the function of these immune cells 6. Through a number of mechanisms, including cytokine inhibitory effects, inhibition of activating receptors, up-regulation of inhibitory receptors, and hypoxia in the tumor microenvironment, the MM microenvironment suppresses the ability of NK and T cells, allowing tumor progression and immune escape for MM cells 7. Researchers have devised numerous novel therapeutic strategies to counteract immunosuppression with the intention of revitalizing the immune defenses against tumors by concurrently activating T cells and NK cells 8. We aimed to review the mechanisms underlying the immunosuppression of T cells and NK cells, explore the common factors contributing to the suppressed state of these immune cells, discuss approaches for dual immune activation, and highlight innovative therapies that synergistically harness the potential of T cells and NK cells in the treatment of MM 9, 10.

## Suppressed immune function of T cells and NK cells

**Figure 1:**
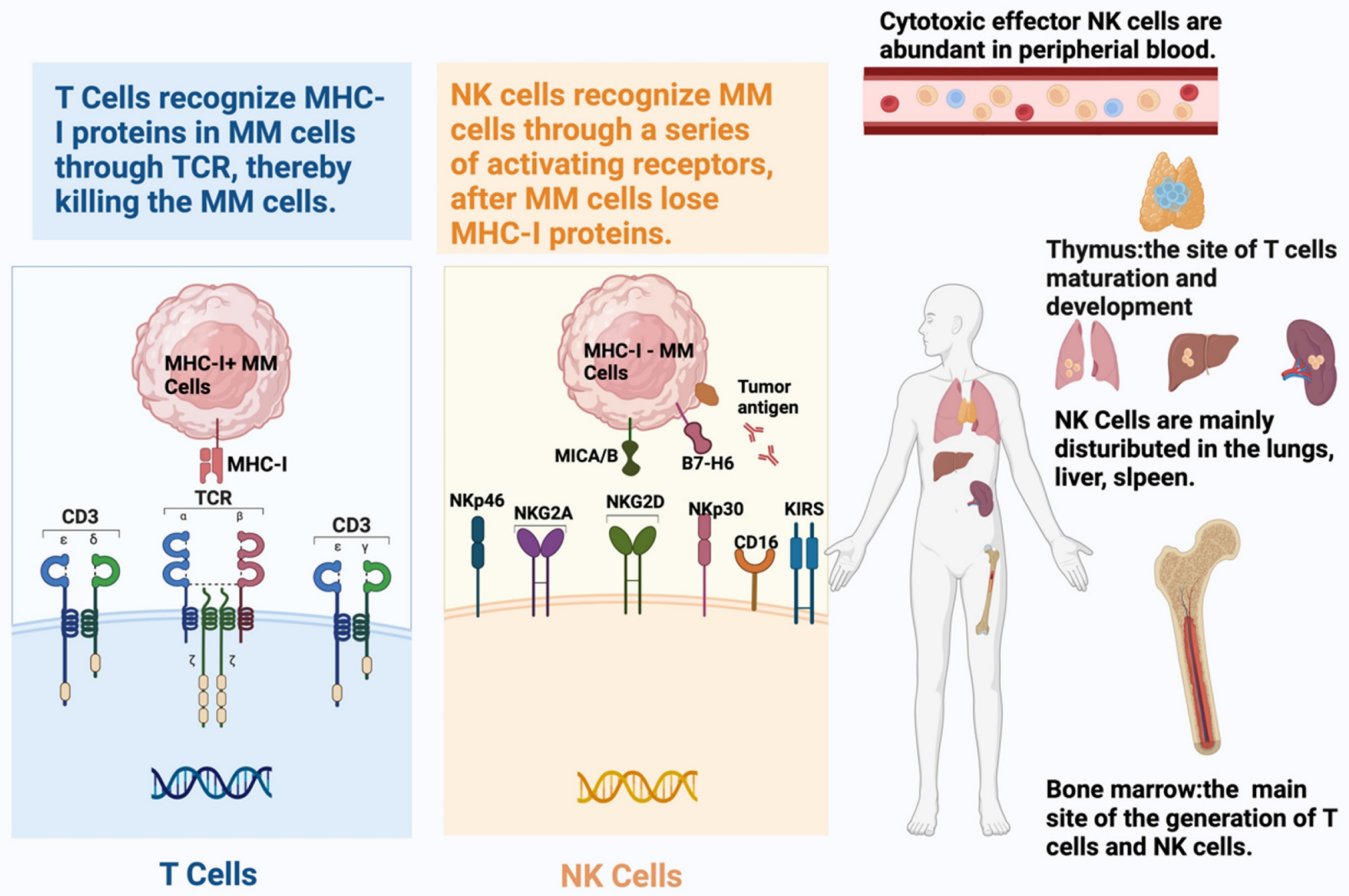
** Schematic representation of the distinct recognition mechanisms and tissue distribution of T cells and natural killer (NK) cells.** T cells recognize tumor cells via T cell receptor (TCR)-mediated binding to peptide antigens presented by major histocompatibility complex class I (MHC-I) molecules, which governs their cytotoxic, cytokine-producing, and proliferative functions. Tumors often evade CD8⁺ T cell responses by downregulating or losing MHC-I expression. In contrast, NK cells employ a repertoire of activating (e.g., NKG2D, NKp46, NKp30, NKp44) and inhibitory receptors to detect stressed or transformed cells, providing a more flexible, non-MHC-restricted mode of target identification.)

The two pillars of adaptive and intrinsic immunity, T cells and NK cells, recognize tumor cells differently (Table 1). As the T cell part of adaptive immunity, CD8^+^ T cells identify tumor cell-derived peptides and eliminate cancer cells through the T cell receptor (TCR), which is displayed by MHC Class I proteins 11. The detection of peptide-MHC ligands can activate T cells, demonstrating the exceptional sensitivity thereof to identify their targets 12. However, this potent recognition mechanism depends on MHC-I protein expression in malignant cells. Therefore, TCRs on CD8^+^ T lymphocytes lose their ability to fight tumor cells if they have mutations or epigenetic changes that result in the absence of MHC-I expression. Contrastingly, tumor cells become targets of NK cells when they lose their MHC-I-like molecules 13. As an important component of intrinsic immunity, NK cells use a variety of surface receptors, including activating and inhibitory receptors, to identify cancerous cells 14-16 (Figure 1).

### T cell exhaustion in MM

**Figure 2:**
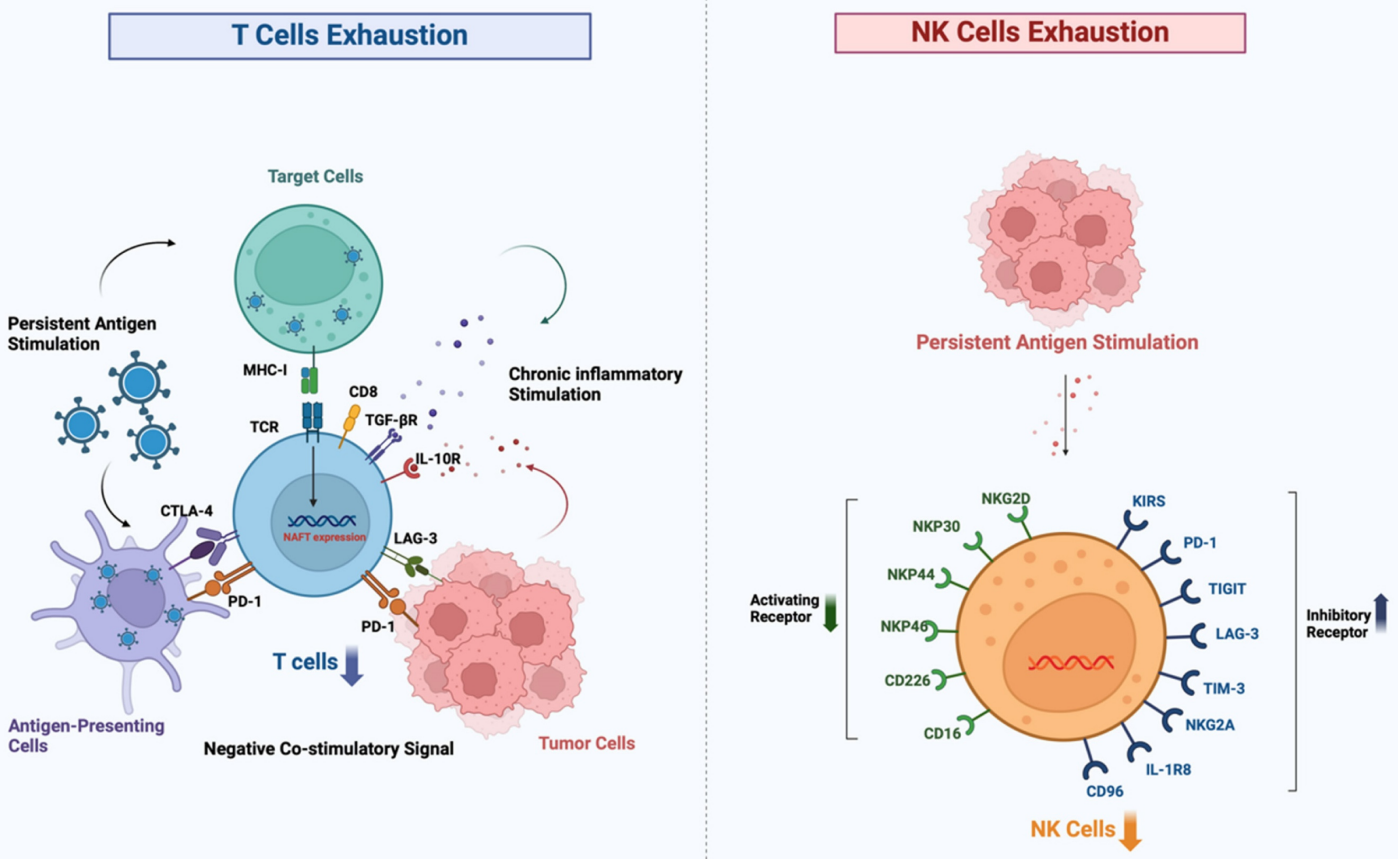
** Progressive exhaustion of T cells and NK cells in the tumor microenvironment.** Under conditions of chronic antigen exposure, such as in cancer, both T cells and NK cells undergo a stepwise functional decline characterized by the upregulation of multiple inhibitory receptors (e.g., PD-1, CTLA-4, TIM-3, LAG-3 on T cells; TIGIT, KIR, NKG2A on NK cells) and the loss of effector cytokine production and cytotoxic activity. This exhausted state enables tumor immune escape and represents a major barrier to effective immunotherapy.)

In MM, the multifaceted nature of T cell suppression can be delineated in various aspects, with T cell exhaustion being the primary manifestation (Figure 2), which is often an unavoidable process that occurs after the effector capacity of T cells diminishes as a result of chronic signaling by the TCR or prolonged antigenic stimulation 17. While this mechanism serves to limit immunopathology under normal physiological conditions, in the context of cancer it paradoxically facilitates immune evasion and promotes the survival of neoplastic cells. Persistent activation of TCR coupled with downstream Ca^2+^ signaling triggers activation of the transcription factor NFAT. This activation enhances the expression of the TOX and NR4A transcription factor families 18. Activation of the NFAT signaling pathway up-regulates the expression of inhibitory immune checkpoint proteins such as CD39, PD-1, LAG3, and CTLA-4, leading to the conversion of T cells with high proliferative and stem cell-like properties into cells that have lost their effector function and replicative potential 19, 20. This process is accompanied by an increase in the expression of inhibitory markers and a decrease in the secretion of inflammatory cytokines 21, 22. Furthermore, an imbalance in the ratio of T cell subsets is also a key feature; in patients with MM, the distribution of T cell subsets is altered. In the early tumor microenvironment of MM, CD4^+^ T cells enhance the cytotoxicity and immune function of CD8^+^ T cells, while CD8^+^ T cells synergize with CD4^+^ T cells to maintain the balance of the CD4^+^/CD8^+^ ratio and maximize anti-tumor effects 23. As MM progresses, there is a noticeable increase in the number of regulatory T cells (Tregs), which inhibit effector T cell activity by secreting suppressive cytokines including transforming growth factor (TGF)-β and IL-10, promoting immune evasion 24. Moreover, a decrease in the percentage of effector T cells hinders the anti-tumor immune response, whereas an increased CD4^+^/CD8^+^ ratio may accelerate the progression of MM 25. Elevated levels of NIMA-related kinase 2 (NEK2) within the tumor microenvironmenT cells is linked to impaired T cell functionality and decreased proliferative potential of CD8^+^ T cells 26. The rapid proliferation of MM cells consumes a large amount of oxygen, leading to the formation of a hypoxic tumor microenvironment where MM cells produce lactic acid through glycolysis under hypoxic conditions 27. In addition, the catabolism of tryptophan to produce urea via Indoleamine 2,3-dioxygenase (IDO) and the production of adenosine via the ectonucleotides CD73 and CD39 are immunosuppressive actions, decreasing the immune function of antitumor cells such as T cells, NK cells, dendritic cells (DC-cells) 28. Treg and suppressor cells derived from MDSCs are recruited in hypoxic environments and secrete immunosuppressive substances that decrease effector cell function 29, 30.

### NK cell dysfunction in MM

**Figure 3:**
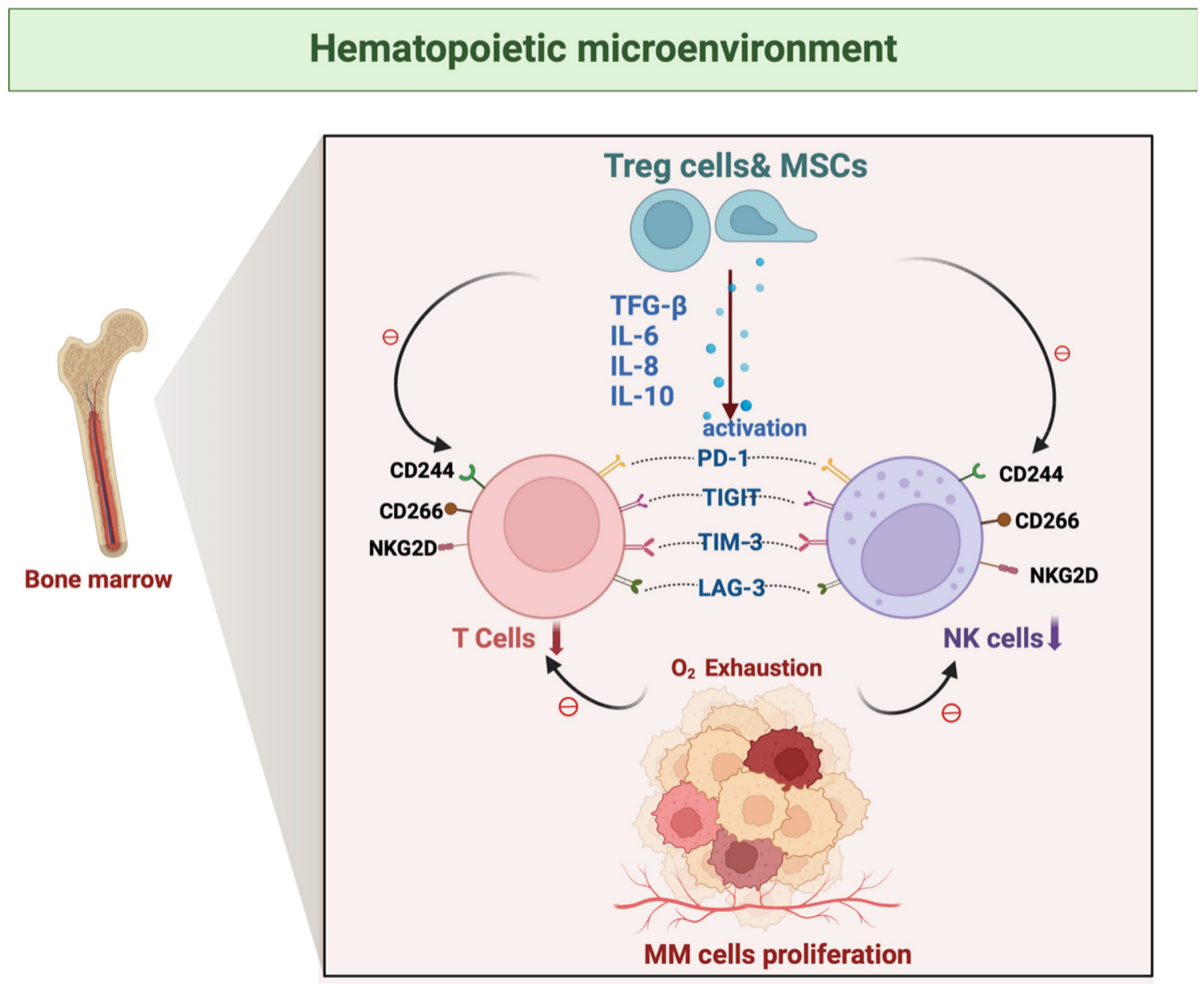
** T cells and NK cells share important activation and inhibition receptor ligand systems.** While T cells and NK cells possess distinct primary recognition systems, they share several critical co-stimulatory and co-inhibitory receptor ligand axes—such as PD-1/PD-L1, TIGIT/CD155, LAG-3/MHC-II, and TIM-3/galectin-9—that are commonly dysregulated in cancer to coordinately suppress both arms of anti-tumor immunity.

The quantity and functionality of NK cells in the peripheral blood of patients are decreasing 31. NK cells kill targets via FasL and TNF-α-related ligands 32. Subsequently, NK cells efficiently eliminate tumor cells through a degranulation-mediated mechanism 33, 34. However, NK cells often exhibit a depleted state in the TME, which weakens the body's immune surveillance function and facilitates the immune evasion of tumor cells 35. The depletion of NK cells is marked by the up-regulation of inhibitory receptors and their corresponding ligands, such as PD-1, LAG-3, TIM-3, TIGIT, KIR, CD96, IL-1R8, NKG2A, and killer cell lectin-like receptor G1, alongside the downregulation of activating receptors and their ligands, including CD226, CD16, NKG2D, NKp44, NKp30, and NKp46^36^ (Figure 3). TME components suppress infiltrating NK cells, in which cytokines such as IL-6, IL-10, and TGF-β can inhibit the anti-tumor activity of NK cells 37. Furthermore, prostaglandin E2 (PGE2) secreted by both tumor-associated mesenchymal stem cells and tumor cells can impair the maturation and cytotoxic functions of NK cells. A crucial player in tumor immunoregulation is the signal transducer and activator of transcription 3 (STAT3), which serves as a pivotal regulator of the transcription of genes responsible for cell growth and differentiation 38, 39. Through stimulation of the STAT3 signaling pathway, tumor cells inhibit the activation receptors NKp30 and NKG2D on the surface of NK cells, while increasing the production of IL-6 and IL-8. Consequently, the activation and anti-tumor functions of NK cells are inhibited. MDSCs hinder NK cell-mediated antibody-dependenT cellsular cytotoxicity. MM cells increase the expression of hypoxia-inducible factor-1α (HIF-1α) in hypoxic environments, which worsens the impairment of NK cell function 40. It has been shown that the exosomal IncRNA NEAT1 derived from multiple myeloma inhibits NK cells and promotes immune escape from tumors by down-regulating PBX1^41^.

## Joint factors of immunosuppression

### Up-regulation of inhibitory receptors

Important suppressor receptors that are frequently overexpressed in the tumor microenvironment and result in immune function suppression are shared by T cells and NK cells, which play key roles in immune surveillance and anti-tumor responses 42 (Figure 3).

l **PD-1**: T cells and NK cells express the inhibitory receptor PD-1, which mostly interacts with its ligands, PD-L1 or PD-L2. Activated and/or fatigued T, B, NK killer, and antigen-presenting cells are the primary cells that express PD-1. In contrast, PD-L1 is expressed in various solid tumors and immune cell subpopulations, indicating both forms of cellular activity. MasT cells, macrophages, and activated dendritic cells are the primary sources of PD-L2 expression 43, 44. The overexpression of PD-1 reduces anti-tumor immune responses, enabling tumor cells to escape immune surveillance. In the bone marrow microenvironment of MM, PD-1/PD-L1 interaction promotes tumor immune evasion and growth. Notably, the expression of PD-1 is markedly upregulated in response to persistent tumor antigenic stimulation, immunosuppressive cytokines such as IL-10, TGF-β, and interferon-γ, hypoxic conditions, and the process of ageing 45.

l **TIGIT**: TIGIT, an inhibitory receptor, is present on both T cells and NK cells and recognizes specific ligands, such as the polyomavirus receptor (PVR) and nectin-2^46^. TIGIT reduces the anti-tumor immune response by binding to these ligands and inhibiting the activation and activity of T cells and NK cells. TIGIT activation also impairs NK cell cytotoxicity and suppresses T cell proliferation 47, 48. TIGIT is widely expressed on the surface of T cells and NK cells in. MM, dramatically reducing NK cell activation and cytotoxicity when it interacts with ligands 49, TIGIT as an immune checkpoint receptor that combines an immunoglobulin-like (Ig) structural domain with an immunoreceptor tyrosine inhibitory motif (ITIM) structural domain, plays a key role in the regulation of immune cell function 50. They can systematically inhibit the effector functions of T cells and NK cells through direct signaling and ligand-mediated indirect pathways. TIGIT significantly impairs NK cell-mediated cytotoxicity by binding to the high-affinity ligand CD155 (PVR), triggering a downstream signaling cascade 51. TIGIT signaling leads to a reduction in the secretion of the pro-inflammatory cytokine IL-12 through modulation of the immune regulatory network of dendritic cells (DCs), while potentially affecting the homeostasis of IL-10 secretion, thus indirectly inhibiting T cells proliferation and NK cells effector function 52. TIGIT indirectly inhibits T cell proliferation and activation. TIGIT directly antagonizes T cell receptor (TCR)-mediated activation signaling by recruiting phosphatases through the ITIM structural domain 53, while also inhibiting T cell proliferation and cytotoxicity, a main cause of immune cell dysfunction 54, 55.

l **TIM-3**: TIM-3, another inhibitory receptor shared withT cells and NK cells, has been found to contain an immunoglobulin-like structural domain (IgV) and a mucin-like structural domain. The IgV structural domain has been demonstrated to bind directly to the carbohydrate-recognition structural domain (CRD) of Galectin-9^56^, which efficiently suppress the activity of both cell types. Furthermore, galactoglucan-9 has been identified as the TIM-3 ligand that specifically recognizes the carbohydrate motif on the TIM-3 IgV^57^. Immune escape is directly linked to the extensive expression of TIM-3 in the cancer microenvironment 57, 58.

l **LAG-3**: In addition to T cells, NK cells also express LAG-3, which reduces the immune response against tumors by attaching to MHC Class II molecules and inhibiting the growth and effector capabilities of T cells and NK cells 59, 60. As another key immunosuppressive molecule on T cells and NK cells, LAG-3 inhibits the activation of T cells and NK cells by interacting with MHC-II molecules 61, weakening anti-tumor effects. In the MM microenvironment, increased expression of LAG-3 further exacerbates the immunosuppressive state 62.

l **CD161**: The CD161 receptor, a member of the C-type lectin receptor family, possesses the ability to form homomeric structures 63. Initially characterized as an inhibitory receptor on NK cells, blocking CD161 augments cytotoxicity against tumor cells expressing the CLEC2D ligand 64. CD161 is also an essential inhibitory receptor in T lymphocytes that infiltrate tumors 65, 66. Several human cancer types express CLEC2D, another C-type lectin receptor, in both tumors and invading myeloid cells. Consequently, therapeutic strategies targeting this inhibitory receptor hold promise for the augmentation of anti-tumor activities of T cells and NK cells 67.

### Downregulation of activating receptors

Activating receptors that share T cells and NK cells are essential for the identification and elimination of malignanT cells or pathogens in the immune system. Multiple variables, such as cytokines, the microenvironment surrounding the tumor, and metabolic conditions, affect the degree of expression and functional activity of these receptors.

l **NKG2D**: The crucial activating receptor NKG2D is mostly expressed on the surface of CD8^+^ T lymphocytes and NK cells 68. Stress-induced ligands, such as MICA, MICB, and members of the UL16-binding protein family, which are commonly found in tumor cells or virus-infected cells, activate immune cells and increase their cytotoxic potential 69, 70. Tumor cells evade immune monitoring by downregulating NKG2D and secreting immunosuppressive cytokines (e.g., TGF-β, IL-10), which inhibit NK and T cell activity 71.

l **CD266**: By down-regulating NKG2D expression and releasing immunosuppressive cytokines like TGF-β and IL-10, which inhibit the activity of both NK cells and T cells, cells of tumors can elude immune monitoring 72. MM cells evade immune surveillance by downregulating the expression of CD112 and CD155, thereby hindering immune cell activation. Three inhibitory receptors, TIGIT, CD96, and PVRIG, oppose the function of CD226. TIGIT and CD96 have a higher affinity for CD155 than for CD226, which leads to rivalry for ligand-binding priority in ligand competition 73.

l **CD244**: Attaching itself to its ligand, CD48 or CD244 —which is an activating receptor found on T cells and NK cells— increases the cytotoxic potential of immune cells 74. In NK and CD8^+^ T cells, CD244 serves as a crucial regulatory molecule. Pro-inflammatory cytokines, including IL-12 and IL-15, potentiate the function of 2B4 and stimulate the activation and proliferation of NK and T cells. However, in certain cancers, tumor cells or tumor-associated macrophages may induce an immunosuppressive microenvironment by modulating the 2B4 signaling pathway and inhibiting its stimulatory effects on NK and T cells 75.

l **CD28:** CD28, an important activating receptor located on the surface of T cells and certain NK cells, facilitates the activation and proliferation of immune cells by delivering co-stimulatory signals upon binding to B7 family ligands (CD80 and CD86) 76. Pro-inflammatory cytokines such as IL-2 enhance T cell and NK cell activation and proliferation by amplifying CD28 signaling. However, CTLA-4 competitively interacts with members of the B7 family, preventing T cell activation and interfering with CD28 binding, eventually modifying CD28-mediated signaling.

l **CD16 (FcγRIII):** NK cells and some T cells have CD16 on the outer surface, which increases the cytotoxic potential of immune cells by attaching to the Fc region of antibodies, promoting the impact of antibody-dependenT cellsular cytotoxicity (ADCC). The ADCC function of NK cells is enhanced by cytokines such as IL-12 and IL-15, which intensify the mediated effects of CD16. In contrast, some cancers can reduce the ADCC activity of T cells and NK cells by altering the characteristics of antibodies or inhibiting CD16 signaling pathways 77.

l **CD137 (4-1BB):**CD137, a co-stimulatory receptor present on activated T cells and NK cells, increases the activity of cytotoxic T cells and NK cells when attached to its ligand, 4-1BBL. This interaction promotes cell survival and proliferation. The stimulatory effect of CD137 on T cells and NK cells can be strengthened by cytokines, such as IL-12 and IL-15, which can also increase CD137 expression. However, tumors can hinder T cells and NK cell lysis by interfering with the 4-1BB signaling pathway or lowering the production of its cognate ligands 78, 79.

### Cytokines

**Figure 4:**
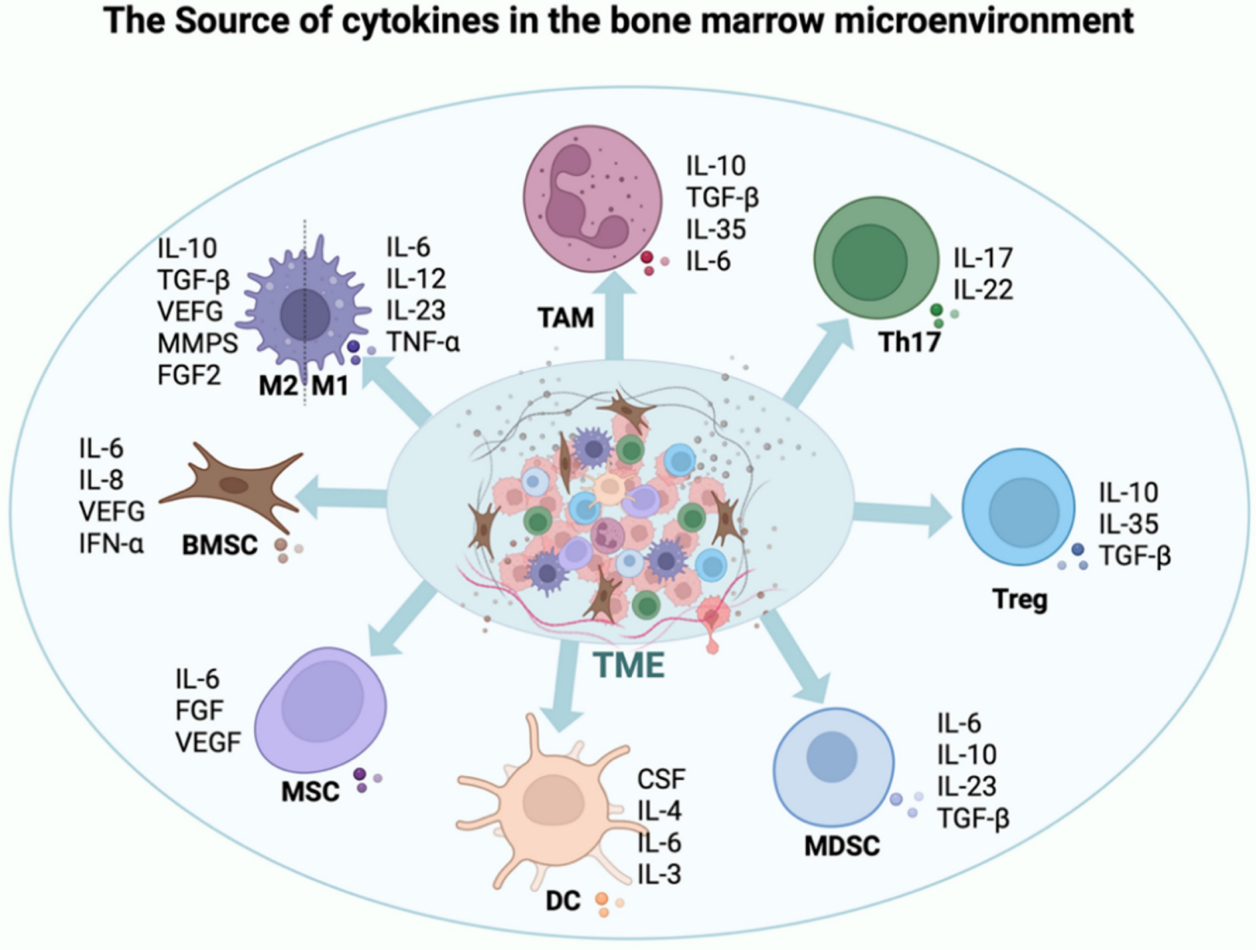
** Cytokine interactions in the MM microenvironment.** This schematic summarizes the key cytokines produced by myeloma cells, stromal cells, and immune cells in the bone marrow niche, highlighting their dual roles in promoting tumor growth (e.g., IL-6, VEGF) and suppressing effector immune cells (e.g., TGF-β, IL-10, PGE2), thereby shaping an immunosuppressive milieu that facilitates disease progression.

In the microenvironment of MM, a diverse array of cytokines plays a pivotal role in inhibiting the functionality of T cells and NK cells. This inhibition worsens tumor growth and aids immune evasion. Myeloma cells and the surrounding immunosuppressive cells, including regulatory T cells and MDSCs, are the main producers of these cytokines 80, 81. They undermine the ability of the immune system to fight tumors through a variety of methods, including inhibiting the release of inflammatory cytokines, promoting the growth of immunosuppressive cells, and downregulating the expression of activating receptors. Therefore, tumor cells can evade immune system monitoring 82, 83. Not all cytokines suppress immune function; for example, IL-18, IL-12, IL-15, and IL-2 enhance T cell and NK cell functions 84 (Figure 4).

l **Transforming growth factor β:** An important immunosuppressive function is played by TGF-β, a cytokine that is prevalent in the tumor microenvironment (TME) of MM. This cytokine controls the fate of differenT cells and alters many metabolic pathways, such as preventing immune cells from infiltrating tumors, preventing anti-tumor immune cells from being activated, encouraging the production of immunosuppressive cells, improving glucose and glutamine metabolism, and interfering with bone metabolism. Additionally, TGF-β inhibits T cell and NK cell proliferation and cytotoxic activity by lowering the expression of activating receptors such as NKG2D 85. In addition, TGF-β promotes the proliferation of regulatory T cells (Tregs), which further exacerbates the immunosuppressive state 86.

l **IL-10:** IL-10, a cytokine that has powerful immunosuppressive qualities, can decrease the anti-tumor potential of T cells by preventing the release of pro-inflammatory cytokines like interferon-gamma (IFN-γ), which inhibits T cell proliferation 87, 88. Simultaneously, IL-10 can inhibit NK cell cytotoxicity and activation, which reduces their ability to kill tumors. Furthermore, by encouraging the growth of Tregs in conjunction with MDSCs, IL-10 intensifies its immunosuppressive effect 89.

l **IL-6**: Through a variety of mechanisms, increased Interleukin-6 (IL-6) expression in MM promotes the growth and survival of myeloma cells and compromises T cells and NK cell functionality. IL-6 can trigger the STAT3 signalling pathway, which inhibits T cell function and lowers the ability to fight tumors. Furthermore, IL-6 reduces the tumoricidal capacity of NK cells by modulating their cytotoxicity 90 91.

l **IL-27**: By promoting the production of Tregs, Interleukin-27 (IL-27) inhibits the growth of T cells, their activity, and NK cell function within the TME. Moreover, IL-27 exacerbates the suppressive effect on NK cell cytotoxicity by inhibiting the release of pro-inflammatory cytokines 92.

l **MSCF**: By modifying the activity of macrophages and suppressor cells derived from MDSCs in the bone marrow, macrophage colony-stimulating factor (M-CSF) indirectly inhibits the function of T cells and NK cells. MDSCs have the ability to release inhibitory cytokines like TGF-β and IL-10, which reduce T cell and NK cell activity and allow the immune escape of malignancies 93.

l **VEGF**: In addition to being essential for angiogenesis, vascular endothelial growth factor (VEGF) exerts an immunosuppressive effect by reducing T cell and NK cell function. VEGF hinders DC cells development, reduces T cell tumor-fighting reactivity, and reduces NK cell tumoricidal potential by altering their activation 94, 95.

l **PGE2**: Prostaglandin E2 (PGE2), an inflammatory mediator, can inhibit T cell and NK cell function in MM by enhancing the production of immunosuppressive molecules such as CTLA-4 and PD-1. Furthermore, PGE2 reduces the cytolytic activity of T cell and NK cells 96, 97.

### Hypoxia

**Figure 5:**
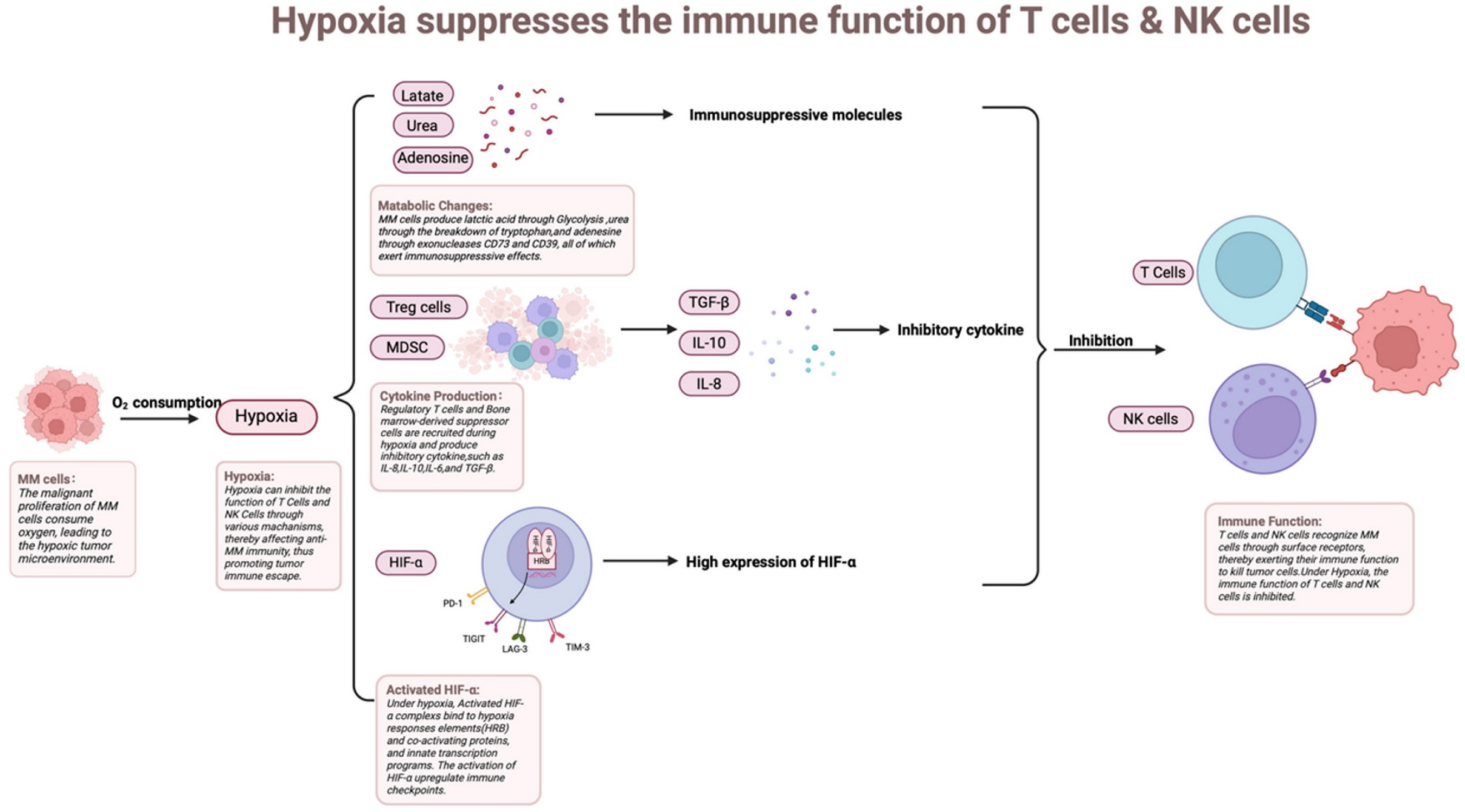
** How myeloma cell proliferation induces hypoxia by depleting oxygen, which in turn triggers metabolic changes, cytokine secretion, and HIF-α activation, ultimately impairing immune cell function.** Rapid myeloma cell proliferation consumes local oxygen, creating a hypoxic niche. This hypoxia stabilizes HIF-α, which in turn reprograms tumor metabolism, induces the secretion of immunosuppressive cytokines (e.g., VEGF, TGF-β), and upregulates checkpoint ligands (e.g., PD-L1), collectively impairing the function and survival of infiltrating T cells and NK cells.

A distinctive feature of the bone marrow microenvironment in MM is hypoxia, which arises from the massive proliferation of MM cells. Hypoxia is strongly associated with tumor development, progression, and resistance to radiotherapy and immunotherapy, and is an important element that contributes to poor patient prognosis 98, 99 (Figure 5). A hypoxic environment can directly or indirectly weaken the immune function of T cells and NK cells, depriving them of effective anti-tumor activity in the TME, which in turn promotes the tumor's immune escape mechanism 100. Under hypoxic conditions, HIF-1α, as a major intracellular response molecule, is able to regulate the expression patterns of a variety of genes 101. Up-regulation of HIF-1α inhibits the function of T cell and NK cells, leading to their depletion or functional failure. Additionally, HIF-1α reduces T cell and NK cell activity by promoting the secretion of immunosuppressive factors (e.g., TGF-β, VEGF, etc.) 102. Hypoxia weakens T cell and NK cell anti-tumor activity, promoting immune escape. This promotes the immunological escape mechanism of the tumor. Through the increased expression of immunological checkpoint molecules on the surface of the tumor and stromal cells, the hypoxic TME promotes immunosuppression. The involved molecules include LAG-3, CTLA-4, PD-L1, PD-1, and T cell immunoglobulin and immunoreceptor tyrosine-based inhibitory motif domain-containing protein TIGIT 103.

### Tumor-associated MSCs

Mesenchymal stem cells (MSCs) with multilineage differentiation potential can differentiate into a variety of cells, such as osteoblasts and chondrocytes, in the BMME of MM. These cells are essential components of the bone marrow stroma, playing a role in the creation of an immunosuppressive microenvironment, and include the release of inhibitory cytokines, direcT cells-to-cell contact, and metabolic regulation 104. MSCs generate large quantities of cytokines such as TGF-β and IL-10 within the microenvironment of MM. These cytokines inhibit T cell proliferation and cytotoxic activity and reduce the lytic function of NK cells by down-regulating the expression of activating receptors on their surface, such as NKG2D. Furthermore, MSCs promote the subsequent generation of regulatory T cells (Tregs), which directly suppress the function of effector T cells to reduce the anti-tumor response and exert an indirect inhibitory effect on NK cells through the secretion of inhibitory molecules such as TGF-β and IL-10^105^.

### Plasma metabolites and platelets

Plasma metabolites promote multiple myeloma (MM) development through metabolic reprogramming and immune microenvironment regulation 106. Tumor cells rely on aerobic glycolysis to produce large amounts of lactate 107, leading to acidification of the microenvironment and enhanced invasiveness, while taking up glutamine, tryptophan and lipids to support proliferation and survival 108. The tryptophan metabolite kynurenine inhibits T cell function and promotes regulatory T cell (Treg) differentiation through activation of the aryl hydrocarbon receptor (AhR) 109, whereas lactic acid and adenosine further diminish cytotoxic T cell (CD8^+^ T) and NK-cell activity 110. In addition, metabolites such as prostaglandin E2 (PGE2) and oxidised lipids promote an immunosuppressive phenotype in myeloid-derived suppressor cells (MDSCs) and M2-type macrophages 111, helping tumors to evade immune surveillance. These mechanisms suggest that targeting metabolic enzymes (e.g., IDO, LDHA) or combining immune checkpoint inhibitors may be potential therapeutic strategies 112, 113.

Platelets play a critical role in multiple myeloma (MM) progression by modulating the immune microenvironment. Studies have shown that platelet activation upregulates immunosuppressive factors such as TGF-β and IL-1^114^, which induces myeloma cells and myeloid cells to highly express PD-L1, and significantly inhibits the anti-tumor activity of CD8^+^ T cells 115. Meanwhile, platelet-secreted chemokines such as CCL5/CCL17 promote the recruitment of regulatory T cells (Treg) in the tumor microenvironment, forming an immunosuppressive microenvironment 116. These immunoregulatory mechanisms promote immune escape and disease progression in myeloma.

## Immunotherapy based on dual immune activation of T cells and NK cells

### Immune checkpoint inhibitors

TIGIT and LAG-3 are critical immune checkpoint molecules that regulate the function of T cells and NK cells. In the microenvironment of MM, the overexpression thereof is closely associated with functional suppression of T cell and NK cells, making TIGIT and LAG-3 viable targets for immunotherapy in MM 117, 118. Monoclonal antibodies against TIGIT release their inhibitory effect on T cells and NK cells by blocking the binding of TIGIT to its ligand CD155, which promotes their proliferation, activation, and cytotoxicity. By blocking these immune checkpoints, T cells regain the ability to recognize and eliminate MM cells while augmenting the direct cytotoxic activity of NK cells. In contrast, LAG-3 monoclonal antibodies disrupt the association between LAG-3 and major histocompatibility complex class II (MHC-II) molecules, restore the proliferative potential and effector functions of T cells, and reduce the inhibitory state of NK cells, thereby increasing their tumoricidal activity. Inhibitors targeting TIGIT and LAG-3 are being evaluated in multiple clinical trials involving solid tumors and hematological malignancies, including MM. Notably, TIGIT inhibitors like tiragolumab, and LAG-3 inhibitors such as relatlimab, have exhibited promising results in enhancing T cell and NK cell function across various clinical settings 119, 120. When administered either as a monotherapy or in conjunction with other immune checkpoint inhibitors, such as PD-1/PD-L1 inhibitors, these inhibitors have demonstrated substantial efficacy in rejuvenating T cell and NK cell activity and decreasing tumor burden. Furthermore, the use of TIGIT and LAG-3 inhibitors in combination with additional immunotherapies, including PD-1 inhibitors, pomalidomide, and chimeric antigen receptor (CAR)-T cells, has exhibited notable synergistic benefits 121. By concurrently targeting multiple immune checkpoint pathways, the suppressive states of T cell and NK cells can be effectively alleviated, thereby markedly augmenting the anti-tumor immune response in patients 122. Significant changes in the immune microenvironment and effectiveness have been observed in patients with MM who received intensive pretreatment after TIGIT and where LAG3 checkpoints were blocked. In the blood and bone marrow, responders in the anti-LAG3 arm have a greater percentage of initial CD4^+^ T cells. Responders in the TIGIT group show higher expression of CD112 in CD8^+^ T cells, whereas non-responders show higher expression of DNAM-1 (also known as CD226) in NK and CD8^+^ T cells, especially in effector memory T cells. By establishing prolonged responses to TIGIT and LAG3 blockade, Dhodapkar et al. made considerable progress regarding the processes behind checkpoint inhibition in MM and revealed new avenues for MM immunotherapy 123.

Much emphasis has been placed on the targeting mechanism of PD-1 monoclonal antibodies and their use in the treatment of MM 124, 125. The bone marrow microenvironment contains PD-1, an inhibitory receptor that is mostly expressed on activated and functionally compromised T and B cells. Furthermore, certain NK and NKT cells were expressed. Tumor cells, immunological cells, and mesenchymal stromal cells all commonly express PD-L1/2 inside the TME 126, 127. When PD-1-positive immune cells bind to these ligands, they recruit and activate protein tyrosine kinases through the immunoreceptor tyrosine switch motif, which triggers a series of signaling transitions, including proximal signaling molecule dephosphorylation, and inhibition of ZAP-70 phosphorylation. This blocks immune cell proliferation and differentiation; reduces immunoglobulin (Ig) secretion; decreases the activity of Ras-MAPK, PKC, and the calcium-calmodulin pathway; limits the release of inflammatory cytokines; and increases the number of Treg cells, weakening the immune response and promoting tumor immune escape, triggering immune suppression and chemoresistance in the organism 128. Inhibition of the PD-1/PD-L1/2 axis rejuvenates the activity of immune cells and augments their anti-tumor capabilities, providing a robust theoretical foundation for immunotherapy in MM 129. PD-1 inhibits the activity of T cell and NK cells by binding to PD-L1 or PD-L2, leading to T cell depletion, reduced proliferation, cytokine secretion, and cytotoxicity. PD-L1, which suppresses anti-tumor immune responses via the PD-1/PD-L1 pathway, is frequently expressed by myeloma and immunosuppressive cells in the tumor microenvironment (such as MDSCs or Tregs) in MM 130, 131. Several PD-1 inhibitors (e.g. navulizumab and pembrolizumab) have been evaluated in clinical studies of MM 132. However, the limited efficacy of PD-1 inhibitors alone in patients with MM may be related to the complexity of the MM microenvironment and the immunosuppressive status of patients.

133. Although early clinical trials demonstrated promising anti-tumor effects of combination therapies, particularly through their ability to significantly enhance T cell immune responses, the landscape shifted in late-stage trials. A notable increase in toxicity risks emerged, especially when these therapies were combined with immunomodulatory drugs, ultimately prompting the FDA to suspend certain trials. 134, 135 This development underscores the critical need for a more meticulous assessment of multiple myeloma (MM) therapeutic strategies, especially considering their complex interactions and potential adverse effects. Concurrently, it is essential to recognize that agents such as lenalidomide and pomalidomide exert multifaceted effects on the tumor microenvironment, including anti-angiogenic and anti-inflammatory properties, modulation of cytokine production, and down-regulation of adhesion molecules 136, which further complicate the therapeutic landscape and necessitate a nuanced approach to treatment evaluation. MM progression correlates with PD-1/PD-L1 upregulation in tumor and immune cells. Although immunotherapies that block the PD-1/PD-L1/2 pathway have been successful in preclinical trials and have demonstrated promising clinical applications, immunotherapies benefit only a subset of patients and have limited efficacy when used alone. Although combination therapy has a high clinical response rate, PD-1 inhibitors are more toxic when used in combination with immunomodulatory drugs, especially in clinical trials, such as KEYNOTE-183 and KEYNOTE-185, where higher rates of serious adverse reactions led to trial suspension 137, 138. The limited clinical efficacy of PD-1/PD-L1 blockade as monotherapy in multiple myeloma, despite compelling preclinical rationale, underscores the unique immunological challenges of the disease 139. The profoundly immunosuppressive bone marrow microenvironment, characterized by abundant regulatory T cells, myeloid-derived suppressor cells, and inhibitory cytokines such as TGF-β and IL-10, may establish a dominant resistance network that is not fully overcome by single-axis checkpoint inhibition. Furthermore, T cells in advanced MM often exhibit a state of terminal exhaustion with co-expression of multiple inhibitory receptors, suggesting that PD-1 blockade alone may be insufficient to restore robust anti-tumor immunity. The significant toxicity observed in late-stage trials combining PD-1 inhibitors with immunomodulatory drugs such as lenalidomide likely reflects an overly amplified and dysregulated immune activation. IMiDs potently enhance T cell and NK cell function through cereblon-mediated mechanisms; when superimposed upon the immune reactivation induced by PD-1 blockade, this can precipitate severe immune-related adverse events, particularly within a bone marrow niche already vulnerable due to disease-related cytopenias and prior treatment. Future strategies to harness the PD-1/PD-L1 axis in MM should therefore prioritize biomarker-driven patient selection and rational combination therapies that concurrently target non-redundant immunosuppressive pathways within the tumor microenvironment—such as those mediated by adenosine, MDSCs, or inhibitory cytokines—rather than relying on broad immunostimulatory combinations. The development of next-generation agents, including bispecific molecules or targeted delivery systems designed to achieve localized immune modulation, may offer a pathway to improved efficacy with a more favorable therapeutic index.

Therefore, the safety and tolerability of combination therapies remain challenging. Drug safety and other issues make immunotherapy of the PD-1/PD-L1/2 pathway remain in an adjunctive position in MM treatment 140, 141, and further exploration and optimization of therapeutic options are needed in the future 140, 141.

### Cytokine-targeted therapies

Several cytokines, including TGF-β, IL-10, and IL-6, help inhibit T cells and NK cells in the complex microenvironment of MM, which makes it easier for tumor cells to elude immune monitoring. Consequently, the development of therapies targeting these immunosuppressive cytokines, or their signaling cascades has emerged as a pivotal strategy to augment immune cell efficacy and revitalize anti-tumor immune responses. TGF-β, a pivotal immunosuppressive agent in the MM microenvironment, promotes immune escape by impeding T cell proliferation and function, as well as diminishing NK cell cytotoxicity. To counteract the effects of TGF-β, researchers have formulated TGF-β monoclonal antibodies, such as fresolimumab, which efficiently prevent the binding of TGF-β to its receptor, thereby mitigating the inhibition of T cells and NK cells 142. Additionally, by inhibiting the kinase activity of TGF-β receptor 1 (TGF-βR1) within the TGF-β signaling cascade, drugs such as galunisertib strengthen the anti-tumor immune response and lessen the suppressive effect of TGF-β on immune cells 143.

The dual blockade of PD-1 and TGF-β rejuvenates the functionality of CD8^+^ T cells in the bone marrow, presenting novel insights into the treatment of MM. An investigation of vactosertib, an inhibitor of TGF-β1 receptor kinase, in conjunction with pomalidomide for the treatment of relapsed MM, demonstrated good tolerability and efficacy 144, 145. Researchers have developed monoclonal antibodies targeting IL-10, such as AM0010 (pegilodecakin), a recombinant derivative of IL-10, which can alleviate immunosuppression and rejuvenate the anti-tumor potential of T cells and NK cells 146. Conversely, IL-6 is abundantly expressed within the MM microenvironment, facilitating tumor cell proliferation and aiding in the evasion of immune surveillance by suppressing immune cell function 146. Hence, interruption of IL-6 signaling is crucial to enhance immune cell functionality. Monoclonal antibodies targeting the IL-6 receptor (IL-6R), such as tocilizumab, effectively prevent the binding of IL-6 to its receptor, thereby decreasing IL-6 signaling and activating T cells and NK cells 147. In addition, JAK/STAT inhibitors, such as ruxolitinib, block IL-6-induced immunosuppressive responses by inhibiting JAK/STAT signaling 148. Conversely, Acoradin exerts an inhibitory effect on MM by suppressing the IL-6-induced JAK2/STAT3 signaling pathway. Although anti-IL-6 therapies have yet to achieve notable success in clinical trials involving patients with MM, their substantial research potential remains and offers a promising avenue for future MM treatment strategies 149, 150.

### Hypoxia-targeted strategies

Within the tumor microenvironment of MM, hypoxia is a prominent characteristic that exerts suppressive effects on the functionality of T cells and NK cells via diverse mechanisms 151. The up-regulation of HIF-1α, a pivotal regulator in hypoxic conditions, has the capacity to modify the metabolic state of the tumor microenvironment, subsequently suppressing the activity of immune cells 152. Drugs and strategies targeting hypoxia-associated pathways are currently under development to activate T cells and NK cells 153. HIF-1α plays a role in promoting immunosuppression in MM by regulating the expression of a series of genes to help tumor cells survive in a hypoxic environment and reduce the activity of T cells and NK cells. The expression level of HIFs is high in patients with MM, and activation of the HIF-related signaling pathway in the hypoxic microenvironment has a significant impact on tumor growth, proliferation, differentiation, and apoptosis, as well as on angiogenesis, MM cell metabolism, immune response, myeloma cell invasion, and metastasis. In response to a hypoxic environment, tumor cells diminish the energy supply of T cells and NK cells through competitive glucose uptake. To restore the function of immune cells, more energy can be provided by modulating glucose metabolism 154. For example, 2-deoxyglucose (2-DG), a glucose analogue, inhibits glucose metabolism and reduces the competition for glucose by tumor cells, thereby enhancing immune cell activity. In addition, STF-31, a selective inhibitor of glucose transporter-1 (GLUT1), inhibits glucose uptake and induces apoptosis in myeloma cells expressing GLUT1^155^. In addition to limiting glucose uptake, studies on inhibition of glycolytic enzymes have also shown potential in MM therapy, such as 3-bromopyruvate (3-BP) targeting hexokinase (HK), which reduces cellular activity and promotes late apoptosis 156. There is a close link between hypoxia and tumor angiogenesis; tumors alleviate hypoxia by promoting angiogenesis, but this process also exacerbates immunosuppression. Therefore, by inhibiting tumor angiogenesis, the hypoxic microenvironment can be indirectly improved, enhancing the function of immune cells 157. For example, pazopanib, an orally available vascular endothelial growth factor receptor-1 (VEGFR-1) inhibitor, had a limited effect in phase II clinical trial in patients with relapsed refractory MM, but is still valuable in improving survival and reducing tumor growth and angiogenesis 158. Sorafenib is also effective in patients with refractory or recurrent MM by targeting the Ras/Raf/MEK/ERK pathway and inhibiting VEGFR-2 and VEGFR-3^159^. Furthermore, the expression and release of endothelin-1 (ET-1) by MM cells plays a role in modulating the hypoxic microenvironment. Macitentan, serving as a dual antagonist of the ET-1 receptor, can disrupt the interplay between the ET-1 axis and HIF-1α, further ameliorating the hypoxic microenvironment by inhibiting the ET-1-activated MAPK/ERK and HIF-1α pathways, thereby augmenting the functionality of immune cells 160.

### Cancer vaccine

**Figure 6:**
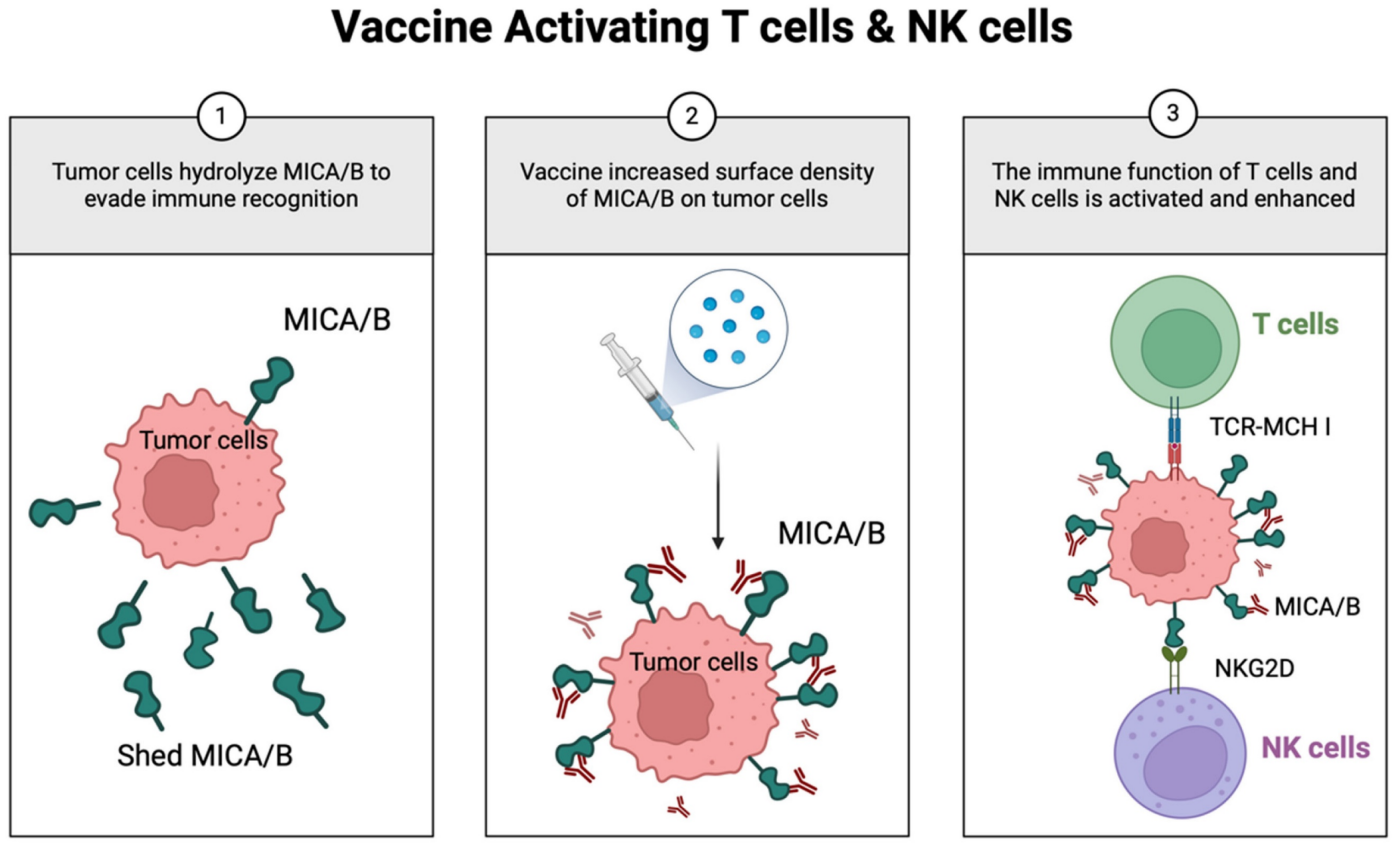
** Novel vaccine simultaneously activates both T cells and NK cells.** MICA/B, a ligand that engages NKG2D receptors on T and NK cells, is often hydrolyzed by tumors to evade immune recognition. The vaccine-induced antibodies inhibit MICA/B hydrolysis, thereby increasing its surface density on tumor cells. This enhances dendritic cell antigen presentation to T cells and strengthens NK cell cytotoxicity.

Scientists are also working on the groundbreaking exploration of tumor vaccines as cutting-edge immunotherapeutic tools aimed at synchronizing the activation of T cells and NK cells to significantly enhance the body's immune response against tumors 161, 162. The research team of Professor Kai W. Wucherpfennig at Harvard University made a breakthrough in this area by developing a novel universal cancer vaccine that innovatively targets MICA/MICB stress molecules to achieve the dual activation of T cells and NK cells. This unique mechanism of the vaccine can help the immune system break through the defense barrier of the tumor and effectively destroy cancer cells (Figure 6). The vaccine overcomes the problem of individual differences in the cancer immune response by inducing a synergistic attack on T cells and NK cells that is not related to tumor antigens, thus demonstrating a powerful synergistic effect. Given that MICA/B expression is widespread in many tumor cells, this choice of target gives the vaccine the potential for broad-spectrum applications, meaning that there is no need for a personalized vaccine tailored to each patient, dramatically improving therapeutic efficacy and feasibility 163. This study reveals a new pathway for immunotherapy in MM and offers a promising therapeutic strategy that is expected to benefit patients with this refractory disease. The extraordinary potential of nano-antibodies to activate T cells and NK cells in tandem has been revealed, which is particularly innovative in therapeutic strategies against hematological cancers such as MM. Here, NK cells and T cells play complementary and intertwined roles in the body's intrinsic and adaptive immune systems, which are critical for the elimination of tumor cells.

### Nano-antibody

**Figure 7:**
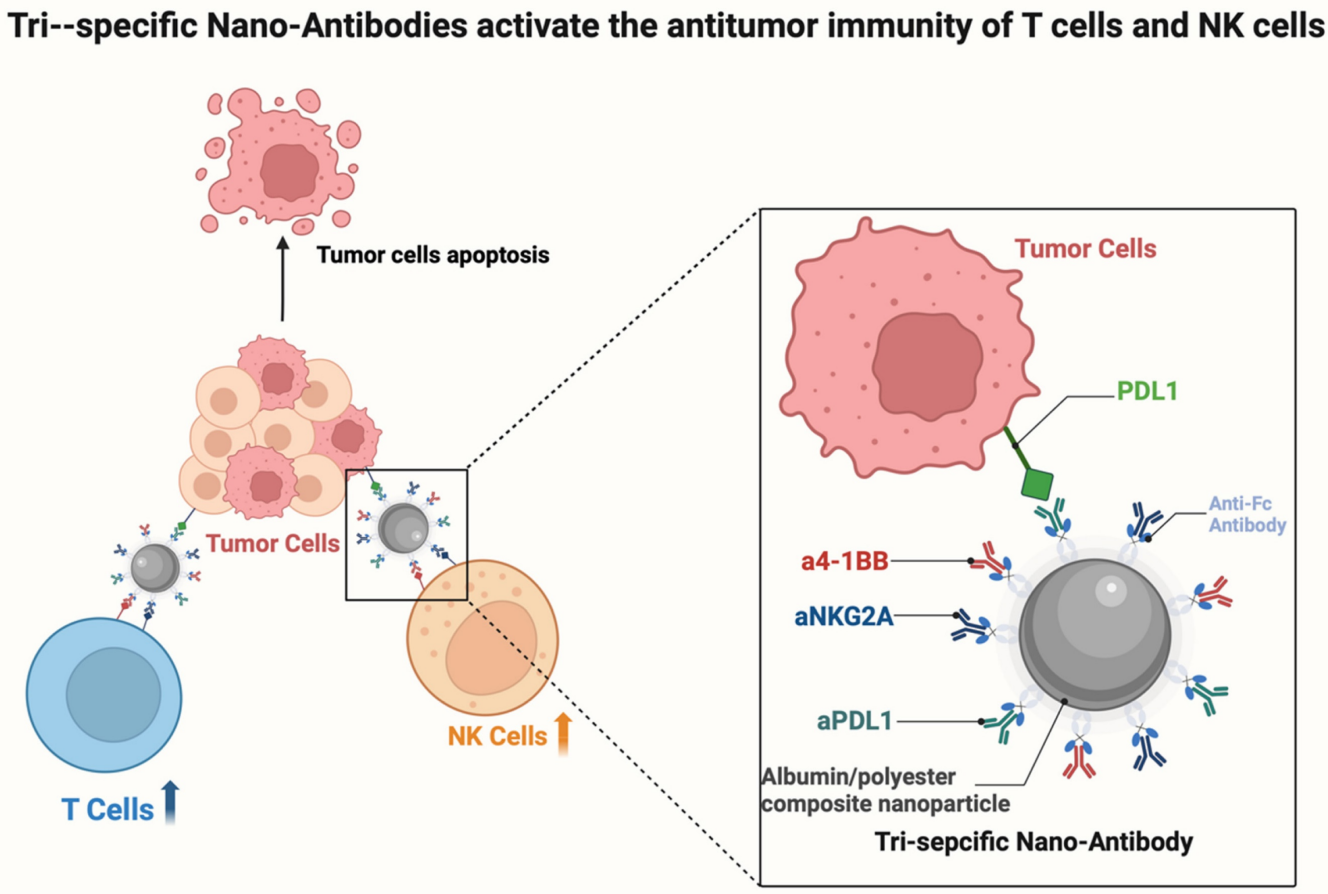
** Tri-Ab simultaneously targets PD-L1, 4-1BB, and NKG2A (or TIGIT) to effectively activate NK and CD8⁺ T cells.** The Tri-Ab molecule simultaneously engages PD-L1 on tumor cells (blocking the PD-1/PD-L1 checkpoint), 4-1BB on immune cells (delivering a co-stimulatory signal), and NKG2A or TIGIT (blocking NK cell and T cell inhibition). This multi-target strategy promotes the synergistic activation, proliferation, and tumor-killing capacity of both CD8⁺ T cells and NK cells within the tumor microenvironment.

Nanoparticles have shown great potential in myeloma treatment because of their high specificity and low immunogenicity. Nano-antibody-based CAR-T cell therapies have achieved remarkable efficacy, which has driven rapid development in this field 166, 167. Researchers at the SCUT, Jun Wang and Song Shen, have demonstrated a method that targets T cells and NK cells simultaneously using multifunctional or nano-antibodies, offering a potential strategy in cancer immunotherapy 164, 165. The group created a novel tri-specific nano-antibody (Tri-NAb) that binds to an anti-Fc antibody and combines three different monoclonal antibodies onto carefully crafted albumin/polyester composite nanoparticles (Figure 7). Tri-NAb exerts anti-tumor effects in multiple myeloma therapy by synergistically activating T and NK cells, but prolonged antigenic stimulation leads to T cells depletion, as evidenced by the upregulation of inhibitory receptors such as PD-1, TIM-3, and LAG-3, and loss of effector function. By simultaneously binding to PD-1, 4-1BB, and NKG2A (or TIGIT), Tri-NAb can efficiently bind to CD8 ^+^ T cells and NK cells. This initiates activation and proliferation, making it easier for the cells to engage with tumor cells and produce better tumoricidal results. By precisely targeting tumor-associated antigens or immunoregulatory molecules, nano-antibodies can augment immune cell function, thereby effectively addressing the intricate immunosuppressive microenvironment of MM. As clinical research progresses, nanobodies are anticipated to pave new avenues for the treatment of MM, offering patients more efficacious and safer immunotherapy alternatives, and shedding new light on the management of this challenging disease 168.

## Dual-targeting strategies in multiple myeloma immunotherapy

Recent advances in multiple myeloma (MM) immunotherapy have yielded promising dual-targeting strategies that enhance anti-tumor efficacy by engaging both T cells and NK cells 173. These approaches overcome key limitations of single-target therapies by addressing antigen heterogeneity and reducing escape mechanisms.

Dual-target CAR-T cells are engineered to recognize two distinct antigens on MM cells, enhancing specificity and potency through dual-antigen engagement. This is particularly beneficial in MM, where antigen heterogeneity is common 174-177. For instance, BCMA/CD38 dual-target CAR-T cells have demonstrated superior cytotoxicity against MM cells in preclinical studies 178, showing enhanced proliferation, cytokine secretion, and tumor infiltration, leading to improved tumor control and survival in mouse models 179. Phase I trial of BCMA/CD38 tandem CAR-T showed 88% ORR in RRMM 180. Preliminary clinical trials suggest this approach is well-tolerated with promising anti-tumor activity.

Bispecific engagers are synthetic molecules designed to bridge immune cells and tumor cells by simultaneously binding to immune cell receptors and tumor antigens 181. This approach leverages the cytotoxic potential of both T cells and NK cells. For example, bispecific T cell engagers targeting BCMA and CD3 redirect T cells to kill BCMA-expressing MM cells 181, 182, AMG 701 (BCMA×CD3) with NK cell-enhanced ADCC showing that ORR was 36% (16/45) at doses 3-12 mg at median follow up of 1.7 months (1-3.7) 183. While bispecific NK-cell engagers targeting CD38 and NKp46 activate NK cells against CD38-positive MM cells. Novel bispecific engagers recruiting both T cells and NK cells are under investigation, aiming for synergistic cytotoxicity and more durable tumor eradication 184, 185.

These innovative strategies represent significant progress in MM treatment. Ongoing research focuses on optimization, safety evaluation, and clinical integration to maximize therapeutic potential.

## Conclusion

Immunotherapy for MM is advancing into a novel phase of development, with a central focus on strategies that elicit a more potent and durable anti-tumor immune response by concurrently activating T cells and NK cells. Remarkable advancements have been made in this field, particularly in the domains of immune checkpoint inhibitors, cytokine modulators, nanobody technologies, vaccine-based therapies, and targeted hypoxic therapies 186-188. Immune checkpoint inhibitors, such as PD-1 and CTLA-4, enhance the anti-tumor activity of T cells by deregulating the tumor suppression of immune cells 141, 189. Conversely, cytokine inhibitors improve the immune microenvironment and promote the activation of immune cells by blocking immunosuppressive cytokines such as IL-6, IL-10, and TGF-β. Nano-antibodies, a new type of biological agent, show great potential to synergistically activate T cells and NK cells simultaneously because of their small size, high stability, and ease of modification. Vaccine therapies aim to generate immune responses against specific tumor antigens by stimulating the immune system, whereas hypoxia therapies target the hypoxic environment inside the tumor to improve the penetration and function of immune cells.

A mechanistic understanding of the synergistic crosstalk and stimulation between T cells and NK cells has become the central rationale for designing next-generation immunotherapies. These findings reveal multiple strategies for overcoming immunosuppression in the MM microenvironment and provide a scientific basis for the development of novel, efficient, and safe immunotherapeutic regimens 190, 191. Despite these advances, future clinical studies face many challenges. Combination therapy, especially that involving a combination of multiple immunomodulators, although theoretically capable of producing stronger anti-tumor effects through synergistic T cells and NK cells stimulation, may also increase treatment-related toxicity and side effects, thereby compromising patient safety and tolerability 192, 193. Therefore, future efforts must focus on optimizing combinatorial regimens based on a deeper mechanistic understanding of T cells and NK cells crosstalk, striving to achieve an optimal balance between amplified efficacy and manageable toxicity.

## Challenges and future perspectives

While simultaneous and synergistic targeting of T cells and NK cells via immunotherapy holds significant promise for MM treatment, several critical challenges must be surmounted to fully harness its clinical potential. Key among these is the management of targeted/non-tumor toxicity, which mandates the development of more specific therapeutic modalities to mitigate off-target effects on healthy tissues. Furthermore, antigen escape mechanisms employed by tumor cells necessitate innovative strategies to counteract tumor heterogeneity and immune evasion. The complexity of manufacturing advanced immunotherapies, such as CAR-T cells, underscores the need for streamlined production processes and cost reduction to facilitate broader clinical adoption. Additionally, the scarcity of reliable biomarkers for predicting therapeutic responses complicates patient stratification and personalized treatment planning. A major remaining gap is the insufficient mechanistic dissection of synergistic stimulation pathways that coordinate T cell and NK cell functions in the MM immune microenvironment. Looking ahead, the integration of combination therapies targeting multiple immune checkpoints offers a synergistic approach to augment anti-tumor immunity, as evidenced by preclinical and early clinical data. The development of cancer vaccines targeting stress molecules like MICA/B, capable of activating both T cells and NK cells, represents a promising strategy to overcome antigen heterogeneity. Moreover, the advent of nano-antibodies, such as Tri-NAb, which can engage multiple receptors to activate immune cells, signifies a technological leap with the potential to enhance therapeutic efficacy while minimizing off-target toxicity. Collectively, overcoming these challenges and leveraging these innovations will be instrumental in advancing MM immunotherapy towards more effective and tailored treatment paradigms.

Despite these advances, several challenges remain. Future research should focus on optimizing combination regimens to enhance efficacy while minimizing toxicity, identifying predictive biomarkers for patient stratification, and exploring novel delivery systems to improve tumor targeting. Additionally, patient-derived organoids and humanized mouse models could facilitate the preclinical evaluation of dual-activation immunotherapies. Ultimately, a deeper understanding of the dynamic interplay between T cells and NK cells in the MM microenvironment will pave the way for more effective and personalized immunotherapeutic strategies.

## Figures and Tables

**Table 1:** The differences between T cells and NK cells

	T cells	NK cells
**Origin**	Hematopoietic stem cells	Hematopoietic stem cells
**Development**	Thymus	Bone marrow, lymph nodes, and spleen
**Recognition**	TCR recognizes antigen peptide MHC complex	NKR recognizes non-MHC-I tumor antigens
**Function**	Adaptive Immunity	Innate immunity
**Surface marker**	CD3, TCR, CD4/CD8	CD16 (FcγRIII), CD56, NKp46, NKG2D
**Inhibitory Receptor**	CTLA-4, PD-1, TIM-3, LAG-3	CD94/NKG2A, KIR, ITIM, PD-1, TIM-3, LAG-3
**Activiatory Receptor**	CD28, CD134, CD137, CD27	NKp44, NKP30, NKP46, NKG2D
**Antigenic Specificity**	Highly specificity	Lower specificity
**Immunologic memory**	Forming memory cells that produce stronger reactions to the same antigen	Not forming memory cells

**Table 2:** The difference between CD38 antibodies, T cell engager, and CART.

	CD38 Antibodies	T cells engager	CAR-T
Representative Drugs	Daratumuma Isatuximab Felzartamab	Teclistamab Elranatamab	Ciltacabtagene Autoleucel Axicabtagene Ciloleucel Tisagenlecleucel
Mechanism	Targets CD38^+^ and exerts cytotoxicity through CDC, ADCC, ADCP effects; activates CD8^+^T cells and CD4^+^T cells for rapid and durable elimination of CD38^+^ immunosuppressive cells	Activates and redirects cytotoxic T lymphocytes to tumor cells to mediate their death by simultaneously binding the CD3^+^ receptor on T cells to BCMA on the tumor cell surface.	T cell expressing CAR molecules recognize tumor antigens on the surface of tumor cells and are activated to specifically kill tumor cells.
Diseases	Multiple myeloma Lymphoma Acute myeloid leukemia Myelodysplastic syndrome Immune thrombocytopenia Autoimmune hemolytic anemia AL Amyloidosis	Multiple myeloma AL Amyloidosis Advanced malignant tumor	Multiple myeloma Lymphoma Acute lymphocytic leukemia Solid tumor
Anti-myeloma efficacy	(Daratumuma)The median PFS and OS were 4.0 months and 20.1 months, the ORR was 31%.	(Teclistamab)The median PFS was 11.3 months., the ORR was 63%	(BCMA-CART) The median PFS was 12.2 months, the ORR was 81.5%
Reference	169, 170	171	172

**Table 3:** The mechanisms, advantages and disadvantages, and products of immune checkpoint inhibitors, cancer vaccine, and nanobodies

	Synergistic stimulation with T cells and NK cells mechanism	Advantages	Disadvantages	Products	Reference
Immune checkpoint inhibitors	Blocks PD1/PD-L1 Blocks TIGIT/CD155 Blocks LAG-3/MHC-II	Dual immune activation Durable immune responses High potential combination therapy Significant synergy with PD-1 inhibitors Reverses immune cell exhaustion	Single drug efficacy is limited Immune-related toxicities Drug resistance Lack of biomarkers Limited clinical data	PD-1 inhibitors: Nivolumab Pembrolizumab Pidilizumab TIGIT inhibitors: Tiragolumab Vibostolimab Domvanalimab Ociperlimab LAG inhibitors: Relatlimab	PMID: 28123899 38229100 28258692 27919908 27269947 31828774 38203720
Cancer Vaccine	Prevents MICA/B hydrolysis MICA/B activates NKG2D receptors	Overcomes tumor heterogeneity Achieves dual activation of T/NK cells	Efficacy needs validation Clinical research is still in its early stages	MICA/MICB targeted vaccine NeoVax	PMID: 35614223 29226910
Nano-Antibody	Multi-target activation	Precise targeting Stable structure Low immunogenicity	Complex manufacturing Preclinical only	Nano antibodies: Tri-NAB	PMID: 39043643 29691900
